# Towards clinical application of freehand optical ultrasound imaging

**DOI:** 10.1038/s41598-024-69826-1

**Published:** 2024-08-13

**Authors:** Fraser T. Watt, Eleanor C. Mackle, Edward Z. Zhang, Paul C. Beard, Erwin J. Alles

**Affiliations:** 1 Wellcome / EPSRC Centre for Interventional and Surgical Sciences, University College London, London, UK; 2 Department of Medical Physics & Biomedical Engineering, University College London, London, UK

**Keywords:** Biomedical engineering, Medical imaging, Ultrasonography, Photoacoustics, Optics and photonics

## Abstract

Freehand optical ultrasound (OpUS) imaging is an emerging ultrasound imaging paradigm that uses an array of fibre-optic, photoacoustic ultrasound sources and a single fibre-optic ultrasound detector to perform ultrasound imaging without the need for electrical components in the probe head. Previous freehand OpUS devices have demonstrated capability for real-time, video-rate imaging of clinically relevant targets, but have been hampered by poor ultrasound penetration, significant imaging artefacts and low frame rates, and their designs limited their clinical applicability. In this work we present a novel freehand OpUS imaging platform, including a fully mobile and compact acquisition console and an improved probe design. The novel freehand OpUS probe presented utilises optical waveguides to shape the generated ultrasound fields for improved ultrasound penetration depths, an extended fibre-optic bundle to improve system versatility and an overall ruggedised design with protective elements to improve probe handling and protect the internal optical components. This probe is demonstrated with phantoms and the first multi-participant *in vivo* imaging study conducted with freehand OpUS imaging probes, this represents several significant steps towards the clinical translation of freehand OpUS imaging.

## Introduction

Optical ultrasound (OpUS) imaging is an emerging ultrasound imaging paradigm that uses light for both the generation and detection of ultrasound, as an alternative to conventional electronic systems based on piezoelectric or capacitive transducer technology. In an OpUS imaging system pulsed light is used to generate ultrasound via the photoacoustic effect^1^ by illuminating an optically absorbing structure at the face of an imaging device^2^. These ultrasound waves propagate into the imaging volume, and back-scattered ultrasound is then detected optically using either a highly sensitive, optically resonant structure^3,4^ or direct optical interferometry^5–7^. Photoacoustic generation of ultrasound in OpUS devices generates wide bandwidths (typically greater than 20 MHz) and high centre frequencies^8^, resulting in probes capable of high resolution ultrasound imaging.

Several different forms of systems have been developed that all employ the OpUS imaging paradigm. Freespace systems, in which OpUS sources are generated by steering a generating beam across a monolithic structure have demonstrated imaging in real time with a range of source geometries^9,10^ and detection methods^5,11^, and have been capable of imaging in realistic tissue-mimicking phantoms phantoms^7^ and *in vivo*^6^. The second form of OpUS imaging systems demonstrated are small, fibre-optic OpUS devices, comprising a single fibre optic transmitter and detector; these probes require rotational^8^, lateral^12^, or scanned^13^ translation to build up an imaging aperture. These devices have been demonstrated to be miniaturisable with diameters below 1 mm, making them suitable for interventional imaging as a standalone instrument^8,14–17^ or for integration into the instrument channels of endoscopes^16,18^.

Recently a new OpUS imaging paradigm has emerged that combines the benefits of both free-space and interventional fibre optic probes: freehand OpUS imaging probes. The freehand OpUS paradigm utilises an array of fibre optic OpUS sources that are sequentially excited and an optical detector, to build up a static imaging aperture and perform synthetic ultrasound imaging. Systems have been reported that have utilised an array of fibre optic OpUS sources, and an electronic receiver, to perform both angle compounded^19^ and synthetic aperture^20^ imaging, however these systems were large and impractical for hand-held imaging. A truly freehand OpUS probe has been demonstrated, comprising an array of multimode optical fibre sources, a monolithic membrane and a fibre-mounted Fabry-Pérot detector^21^. This system was used to perform real-time, video-rate OpUS imaging of small fields of view, in a form factor that resembled that of a conventional electronic handheld probe. The imaging quality achieved with this probe was negatively impacted by a low number of sources and a small, circular, source shape that caused rapid geometrical dissipation of pressure away from the imaging plane, resulting in significant image artefacts and limited imaging depth. In addition a frame rate of 11 Hz was achieved which is still significantly slower than more technologically mature electronic ultrasound probes.

In this work a new freehand OpUS imaging system is presented that incorporates multiple design elements to address the issues outlined above. The presented OpUS probe utilises eccentric optical waveguides to shape the output from an array of optical fibres and generate an array of elliptical OpUS sources that, when compared to symmetric sources, generate an extended, collimated near field zone in the elevational direction, providing greater imaging depths and improved B-scan SNR when compared to previous designs. The OpUS probe head was connected via an extended, 11-meter long fiber bundle to provide flexibility for use in a range of imaging scenarios both in the laboratory and clinically-relevant settings. Specifically, this device was designed to target multimodal imaging applications, with the length of the fibre bundle required to achieve in-bore multimodal imaging alongside magnetic resonance or computed tomography imaging systems. The novel OpUS probe design was combined with a bespoke, mobile, OpUS imaging console that could provide real-time freehand OpUS imaging speeds of up to 24 Hz, a significant increase in imaging speed compared to other freehand OpUS systems. The acoustic properties and imaging capabilities of the probe are demonstrated, and the system is used for imaging experiments with both a tissue-mimicking imaging phantom and in a multi-participant *in vivo* imaging study.

## Methods

### Freehand OpUS system: overview

**Figure 1:**
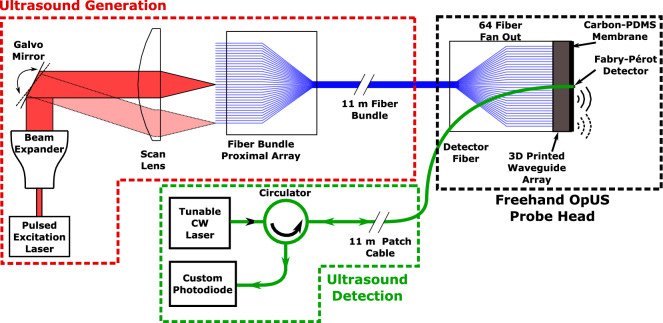
Schematic of the freehand OpUS acquisition system. The pulsed excitation light is focused and sequentially coupled into the flat-cleaved ends of a 64-fibre bundle by rapid scanning mirrors, where a beam expander was added to improve the focal quality. The flexible bundle is then fanned out on an acrylic substrate (Fig. 2c,d) and butt-coupled to an array of 3D printed, eccentric, optical waveguides to shape the fibre output. Ultrasound detection is achieved by continuous monitoring of the reflectivity of a fibre-mounted Fabry-Pérot cavity positioned centrally in the OpUS source aperture.

Figure 1 provides a schematic overview of the freehand OpUS acquisition system presented in this work. The freehand OpUS probe comprises an array of 64 optical fibres, an array of optical waveguides to shape the output from the optical fibres and an absorbing membrane at the distal end. To conduct ultrasound imaging with this probe, a pulsed laser light source is scanned across the proximal end of the fibre array, sequentially exciting OpUS fields via the photoacoustic effect in the absorbing membrane and the resulting fields are transmitted into the imaging target. Reflected ultrasound waves are detected by a single fibre optic ultrasound receiver mounted in the probe head. As each fibre in the source array is coupled into in turn, a pulse-echo imaging aperture is built up, with each channel corresponding to a single OpUS source in the array.

In this system, the beam from a pulsed excitation light source (wavelength 1064 nm; pulse duration: 1.5 ns, pulse repetition rate: 2.7 kHz; maximum pulse energy 71 µJ; DSS1064-Q3, Crylas Germany) was manipulated by rapidly scanned galvanometer (galvo) mirrors (GVS002, Thorlabs, Germany) controlled via a data acquisition card (NI USB-6211; National Instruments, Tx, USA). The beam was then focused by a scan lens (focal length: 110 mm; field of view: 28.9 mm × 28.9 mm; LSM05-BB, Thorlabs, Germany) and rapidly switched between the discrete tips of a custom manufactured fibre-optic bundle. The galvo mirror motion was set to move between fibre tips in a stop-start fashion, with the overall speed limited by the pulse repetition rate of the laser; this equates to a maximum traversal rate of 42 Hz for all 64 fibres in the OpUS probe used in this work. Back-scattered ultrasound was detected by a fibre-optic Fabry-Pérot sensor, which was interrogated by a tunable continuous-wave light source tuned to the wavelength corresponding to maximum sensitivity for the cavity^3^ (wavelength range: 1500 - 1630 nm; TUNICS T100S-HP, EXFO, Canada), delivered to the cavity via a circulator (6015-3-APC, Thorlabs, Germany). Back-scattered ultrasound would impinge on the cavity and modulate the cavity thickness, thereby altering the reflectivity of the cavity, which was interrogated using a custom photodiode. The DC component of the photodiode signal (0-500 kHz) was used to maintain sensor sensitivity, whilst the high-frequency component (> 500 kHz) contained the pulse-echo signal. Ultrasound B-scans were acquired through sequential recordings of pulse-echo time traces for each fibre-optic source, with optional averaging performed by delivering multiple laser pulses to the same source without moving the galvo mirrors.

**Figure 2:**
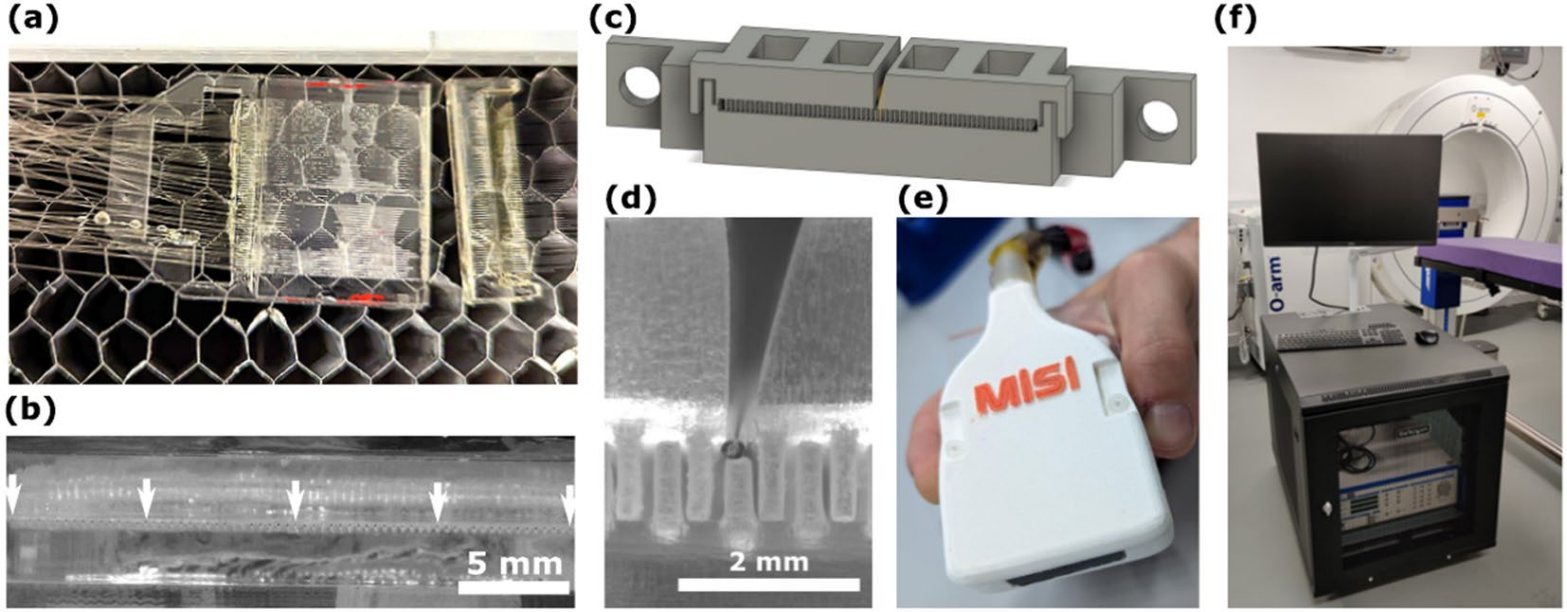
The Freehand OpUS Imaging System (**a**) The fibre optic array in the laser cutter bed after cutting. Both the main substrate of the waveguide array and the overhanging extra fibre that was removed are visible. The array was subsequently manually polished. (**b**) High magnification image of the distal end of the fibre optic bundle after manual polishing, prior to coupling to the waveguide array, demonstrating the regular fibre pitch, the position of every 16th fibre tip has been marked. (**c**) rendering of the model for the 3D printed eccentric waveguide array that forms the front face of the freehand OpUS probe. The waveguide array is printed in three pieces, the bottom 32 elements are printed on a solid frame, and the top 32 elements are printed in two halves to facilitate the placement of the Fabry-Pérot detector fibre. (**d**) High magnification image of the central elements of the eccentric waveguide array, with a detector fibre positioned above the central, shortened waveguide. This image confirms the rectangular shape and interlocked patterning of the waveguides, and the placement of the detector within the elevational extent of the source aperture. (**e**) The full freehand probe head, encased in an ergonomic protective clamshell. Also visible are the OpUS generating membrane and a 3D printed strain relief boot protecting the fibre-optic bundle. (**f**) Image of full freehand OpUS imaging console in operation configuration, with monitor attached.

### Freehand OpUS imaging probe

A novel design for a freehand OpUS imaging probe was developed, which included a number of features to facilitate its deployment outside of the laboratory environment, a key element of this being a flexible and versatile fibre bundle, long enough to be used in a range of experimental scenarios. To achieve this, a custom, 11 m long bundle comprising 64 multimode optic fibres (core diameter: 105 µm; FG105LCA, Thorlabs, Germany) was fabricated. The proximal end of the fibres were stripped and flat-cleaved, and set in two rows on an acrylic substrate, with 400 µm pitch and a 5 mm overhang beyond the substrate edge to prevent optical damage from the excitation laser light to the acrylic or adhesive used to bond the fibres. The distal end of the bundle required all fibres to be terminated in a flat array to enable coupling into further optical elements. To prevent damage and alignment inaccuracies induced in stripping and cleaving the number of fibres required, a method for laser cleaving the full fibre array was developed. An acrylic holder was fabricated, comprising an engraved substrate to guide and hold the fibres and a second flat piece of acrylic that was placed on top of the substrate and clamped in place, with the only space between the two acrylic pieces being the engraved grooves for fibres. The cladding of each fibre was stripped across the length of the acrylic substrate and the stripped fibre was then fed through one of the engraved grooves between the two acrylic pieces. Provided that the stripped portion of the fibre was long enough to extend over the entire substrate, the actual length of fiber stripped was not important, reducing the required accuracy in fibre stripping. Ideally, the fibres would not be stripped at all, however initial trials demonstrated that the adhesive available would not bond effectively to the fibre cladding. Once all fibres were held in place, UV-curing adhesive (NOA81, Norland Products, NJ, USA) was then used to fill the spaces around the fibres, filling the engraved grooves around the fibres via capillary action, before being cured. The full fibre array was then laser cut with a single cut (Fig. 2a), removing the overhanging fibre. To improve the array flatness and optical coupling efficiency, the face of the fiber array was manually polished, resulting in a monolithic acrylic and adhesive block with a flat array of polished fiber tips embedded in it (Fig. 2b). To protect the length of the fibres, the bundle was wrapped in a PET cable sleeve (diameter: 6.35 mm; G1301/4 BK007, Alpha Wire, NY, USA).

The earlier freehand OpUS probe developed by Alles *et. al.*^21^ comprised an array of circular fibre-optic OpUS sources with small diameters (*ca.* 200 µm), and as a result the generated OpUS fields demonstrated rapid geometrical attenuation, which limited ultrasound pressures in the imaging plane, and hence achievable imaging depth. Previously it has been shown^9^ that the OpUS penetration depth can be improved by generating eccentric ultrasound sources that extend significantly in the elevational dimension to limit associated geometrical spreading. Previously, various means of achieving eccentric OpUS sources have been explored^22^, where direct 3D printing of waveguides was found to be the most reproducible and scalable. As such an array of 64 3D printed eccentric waveguides was fabricated, to shape the output from an array of fibre optic light sources. Each waveguide had dimensions 200 µm (lateral) by 1 mm (elevational) by 10 mm (long), and the array was printed at 400 µm pitch, resulting in a final array width of 25.2 mm. To facilitate 3D printing without warping, the array was printed as two arrays with 32 elements each at 800 µm pitch, which would then be interleaved to form the finished array. To ensure that the detector sat centrally in the imaging plane and located within the elevational extent of the source aperture, the central waveguide in the bottom array was printed at a reduced height of 800 µm, and the top array was printed in two halves to generate a gap in which the detector could sit. A 3D rendering of the full waveguide array, and an image visualising the waveguides and the positioning of the detector fibre are seen in Fig. 2c,d. An optically clear resin (RS-F2-GPCL-04, Clear Resin V4, Formlabs, MA, USA) was used to print the waveguides at 25 µm layer height on a consumer-grade stereolithography (SLA) printer (Form 3, Formlabs, MA, USA). The waveguide array was assembled and set in place with a low refractive index UV-cured adhesive (published refractive index: 1.46; NOA85, Norland Products, NJ, USA) to act as optical cladding around the waveguides; the end faces were then manually polished and an absence of optical cross-talk was confirmed through white light through-illumination. Accurate optical coupling of excitation light into the waveguide array was facilitated by a 3D-printed coupling tray to hold the the distal end of the fibre bundle in place and butt coupling each fibre to a single waveguide. For OpUS generation, an elemental carbon-loaded polydimethylsiloxane (PDMS) membrane^7^ of 25 µm thickness was prepared and bonded to the distal face of the waveguide array with uncured, carbon loaded PDMS. Once cured an incision was made in the membrane for the placement of a fibre-optic detector, and the edges of the membrane were sealed with UV-curing adhesive (NOA81, Norland Products, NJ, USA), to reduce the risk of the membrane tearing due to mechanical contact during freehand imaging or water seeping under the membrane when imaging in a water bath.

Ultrasound signals were detected by a fibre-mounted plano-concave Fabry-Pérot cavity mounted at the tip of a single mode optical fibre that was positioned in a groove fabricated along the top of the waveguide array (Fig. 2d). Previous work has demonstrated that fibre-optic detectors of this type are effectively omnidirectional up to 35 MHz^23^, covering the typical bandwidths of OpUS sources. To ensure that no signal was blocked the fibre tip was left protruding by approximately 2 mm to ensure back-scattered ultrasound signal was not blocked by the edges of the waveguide array. The fibre-optic detector was friction-clamped rather than bonded to the acrylic substrate to facilitate replacement in case of damage. Finally, the detector fibre was fusion-spliced to an 11 meter pig tail, with the splice point protected by a custom 3D-printed polymer splint, and the pigtail was fixed to the outside of the PET sleeve protecting the fibre bundle.

The active elements of the probe head were enclosed in a polyactic acid (PLA; Pearl White PLA, 1620, Ultimaker, Netherlands) clamshell with approximate dimensions 60 mm (width) by 21 mm (height) by 91 mm (length), approximating the size of a conventional electronic probe (Fig. 2e). The edges and corners of the shell were rounded and sanded smooth to ensure safety and comfort when brought into contact with an imaging target. To prevent damage to the fibre bundle during freehand manipulation, strain relief pieces were fabricated in an elastic resin (FLELCL01, Elastic Resin 50a V1, Formlabs, MA, USA).

### Data acquisition and processing

To facilitate deployment in a range of environments, a mobile OpUS acquisition console was assembled (Fig. 2f). All components required for OpUS imaging were mounted inside an enclosed and mobile 19-inch server rack, forming a console of approximately the same size as a conventional ultrasound imaging console.

System control and data processing were performed with a rack-mounted blade PC (Precision 3930 Rack, Dell Corporation, Tx, USA; CPU: Intel Core i7-8700K, RAM: 128 GB) with an internal data acquisition card (sampling frequency: 250 MHz; bit-depth: 16 bits; M4i.4420-x8, Spectrum, Germany) and graphical processing unit (GPU; Quadro P6000, NVIDIA Corporation, CA, USA).

Ultrasound B-scans were acquired through sequential recordings of pulse-echo time traces for each fibre-optic source, with optional averaging performed by delivering multiple laser pulses to the same source without moving the galvo mirrors. The B-scan was band-pass filtered (cut-on: 2 MHZ, cut-off: 12 MHz), and then passed to a GPU-accelerated beamformer^24^ for image reconstruction, followed by envelope detection and log-compression. To maximise the imaging frame rate, data acquisition of the current frame, reconstruction of the preceding frame, and optional saving of the pre-preceding frame were performed in parallel. The system was capable of performing real-time, continuous, reconstructed imaging at a maximum frame rate of 24 Hz in absence of averaging. This provided suitable image quality for highly echogenic targets, however, depending on the echogenicity of the object a varying number of averages was set. Ten-fold averaging was used for weakly echogenic wire targets, resulting in a frame rate of 3.2 Hz. For soft targets (i.e. tissue-mimicking phantoms or *in vivo* imaging), three-fold averaging was applied at a frame rate of 10.1 Hz.

### Probe characterisation and performance testing

The acoustic performance of the freehand OpUS probe was characterised with a calibrated needle hydrophone (calibrated bandwidth: 1-30 MHz; diameter: 200 µm; Precision Acoustics, UK) mounted on a pair of orthogonally-mounted motorised stages (MTS50/M-Z8, Thorlabs, Germany) and moved in a plane parallel to the face of the OpUS probe to acquire an ultrasound field scan. Independent field scans were taken for each source across a 30 mm (wide) by 3 mm (high) field at 0.01 mm step size in both directions, measured at 1.5 mm from the probe face. Due to the extended nature of the field scan (*ca.* 54 hours), this was conducted at low laser power (20%; pulse energy: 14.2 µJ) to prevent burn-in damage to any optical component. This scan was then used to give an estimation of the location and dimensions of the sources, assess inter-element cross talk and analyse the shape of the OpUS fields as they propagated into the imaging volume. To assess the ultrasound pressures under normal operating conditions, a single point measurement of each source was taken with the same needle hydrophone positioned at the point of maximum signal for each source, with full laser power (pulse energy: 71.4 µJ).

The imaging performance of the probe was assessed by imaging of a scanned wire target, acting as a point scatterer. A single tungsten wire (diameter: 27 µm) was placed orthogonal to the image plane and submerged in water; the wire was then scanned with orthogonal motorised stages (MTS50/M-Z8, Thorlabs, Germany) across a 16 mm (lateral) by 20 mm (axial) area at 1 mm steps in both directions, and OpUS imaging was conducted at each point. The lateral and axial resolutions were then extracted for each wire position. To assess the performance variation with depth, a wire phantom consisting of 15 tungsten wires (diameter: 15 µm) spaced 2 mm apart was positioned and imaged along the axial axis.

### Phantom imaging

A tissue mimicking phantom was used to assess the capability of the probe to image structures with geometries and ultrasound contrasts similar to that seen in clinical imaging. The phantom was fabricated from a tissue mimicking material of 10% w/w poly(vinyl) alcohol cryogel^25^, and held two embedded, wall-less cavities of approximately 2 mm diameter, modelled after blood vessels. For imaging, the phantom was submerged in deionised water and the probe was fixed approximately 5 mm above the phantom surface. As a demonstration of video-rate imaging, freehand OpUS video was recorded as one vessel in the phantom was flushed with a 10% w/w mixture of glass spheres and water acting as a contrast agent, providing a dynamic phantom with time-varying contrast profile.

### Human volunteer imaging study

As a further step towards clinical translation, a multi-participant *in vivo* study with five healthy volunteers was carried out. Two imaging targets were used in the study; the first target was imaging of a finger in a waterbath, to mimic some of the controlled conditions used in phantom imaging but with an *in vivo* imaging target. The hand of the volunteer was submerged and cross sectional imaging of the index finger between the distal and proximal interphalangeal joints was performed, with the participant moving their hand relative to the probe surface whilst video-rate OpUS data were continuously recorded. The second imaging target was the common carotid artery on the right hand side of the neck. A small amount of clinical coupling gel was applied between the probe face and the skin, and the probe was manually manipulated to identify the structures in the neck. Both imaging targets were also imaged with a conventional piezoelectric freehand ultrasound probe (active wavelength range: 2-6 MHz; L15 HD3, Clarius, Canada). The *in vivo* study described was approved by the University College London Research Ethics Committee (project ID: 17299/001), where the study was conducted. All experimental protocols and methods were carried out in accordance with relevant ethical guidelines and informed consent was obtained from all participants.

**Figure 3:**
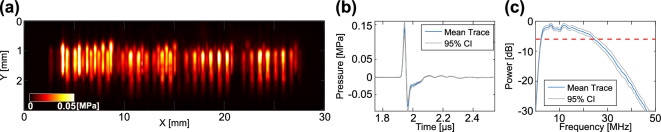
Acoustical characterisation of the freehand OpUS probe (**a**) Composite field scan comprising the coherent summation of field scans for each OpUS source in the array after back-propagation to the transducer surface, pressure presented in MPa. (**b**) Mean and 95 % confidence interval of the 61 time traces measured on-axis at full laser power, measured at 1.5 mm from the generator surface. (**c**) Mean and 95 % confidence interval of the acoustic power spectra for all 61 OpUS sources in the freehand probe.

**Figure 4:**
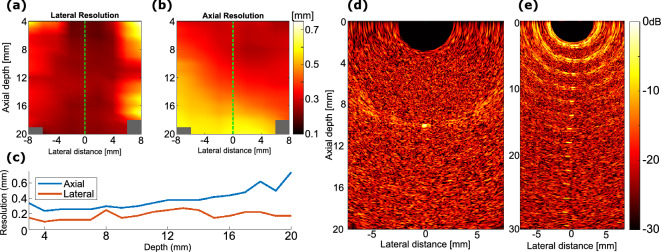
Resolution maps and wire phantom images collected for the freehand OpUS probe. (**a**) Lateral resolution map. (**b**) Axial resolution map, (a,b presented on the same color scale). (**c**) Measured resolutions along the lateral centre of the imaging plane (marked on (a and b)). (**d**) DaS-reconstructed, freehand OpUS image of a single tungsten wire (diameter: 27 µm, acting as a point scatterer) located approximately centrally in the imaging plane, collected at 3 averages. (**e**) DaS-reconstructed, freehand OpUS image of fifteen tungsten wires (diameter: 15 µm) arranged at 2 mm intervals to 30 mm imaging depth. (**d**) and (**e**) are presented on the same logarithmic scale.

## Results

### Acoustical performance

The field scan of each source was back-propagated to the generator surface using the angular spectrum approach (ASA)^26^, the field for each source was then coherently summed to assess the overall pattern of the OpUS source distribution in the freehand probe (Fig. 3a). The shape of the resulting composite field scan closely follows that of the waveguide array, when inspected individually each of the field scans confirmed an absence of optical and acoustical cross-talk between the discrete sources. When propagated forwards using the ASA method, the probe exhibited an average beam dimension (at the -6 dB level) of 1.2 ± 0.2 mm (lateral) by 1.3 ± 0.2 mm (elevational, mean ± standard deviation) at a depth of 5 mm, and 2.0 ± 0.4 mm (lateral) by 1.8 ± 0.3 mm (elevational) at 10 mm. The corresponding elevational resolution is both smaller than in previously reported systems, and increases at a lower rate with depth^21^, confirming that the eccentric sources improve confinement of acoustic energy to the image plane, when compared to circular sources.

In addition, the individual field scans revealed that three of the fibres in the array were damaged and would not guide light to the waveguides, and as such would not generate ultrasound. On inspection of the distal end of the bundle, these fibre breakages were identified as having occurred during the process of setting the fibres in place prior to laser cutting, as the damage was confined to points within the acrylic substrate, but away from the laser cut edge. The damage occurred during the manual handling of the fibres required in the fabrication process, and could be avoided in future designs by the use of pre-fabricated bundles. All further imaging was conducted with 61 sources in the array. Single point needle hydrophone measurements at full laser power (Fig.3b,c) demonstrated an average pressure generation peak of 0.14 ± 0.08 MPa (mean ± standard deviation), with a peak pressure of 0.3 MPa. These pressures are in line with other OpUS devices with fibre optic sources, despite a source area that is approximately six times greater than in previous work^21^, which confirms more efficient optical coupling through the imaging probe as a result of the inclusion of the waveguides and from the improved galvo mirror scanning system. To compensate for the spatial variation in generated pressure values, the 61 point pressure measurements were used to generate a scaling vector to equalise recorded B-scans in imaging and compensate for variable pressure generation. The average bandwidth generated was 18.6 ± 2.3 MHz at the -6 dB level with a centre frequency of 10.8 ± 1.2 MHz, in line with ultrasound generation from typical OpUS sources^2^ and highly homogeneous across the array, confirming the uniform performance across the PDMS membrane.

### Imaging performance

Imaging of a thin tungsten wire acting as a point scatterer at varying position was used to generate maps of the imaging resolution across the imaging plane (Fig.4a, b). The axial resolution of the probe demonstrated a significant decrease at depth in the left hand side of the imaging plane as a result of the variation of performance to the right of the source array (x > 20 mm in the field scan) and the collocation of the non-functional elements. The reduction in pressure generation to one side of the array may have arisen from reduced optical coupling between the fibre bundle and waveguide array on one side, due to the distal end of the bundle sitting at a slight angle relative to the waveguide array. The asymmetric source strength may also have generated the asymmetry in lateral resolution shown in Fig. 4a, the stronger sources on the right hand side of the image (left-hand-side of the array as oriented in Fig. 3a) result in a wider “effective aperture” – and corresponding higher resolution–experienced by objects positioned in the left half of the image. Acoustic cross-talk generated by this probe arising from the detector protruding slightly from the face of the probe and the reduced signal received close to the probe due to the directional nature of the OpUS sources, prevented measurement of the resolutions below 3 mm, this can be seen at low axial depths in Fig.4e. Measured at an axial depth of 5 mm (Fig 4c,d) the lateral resolution (125 µm) compared favourably to that of a previously presented system (169 µm^21^). However, the axial resolution (260 µm) was lower than previously reported (173 µm); this was a result of a higher bandpass filter cutoff being used (12 MHz in this work, 7 MHz in previous work^21^).

**Figure 5:**
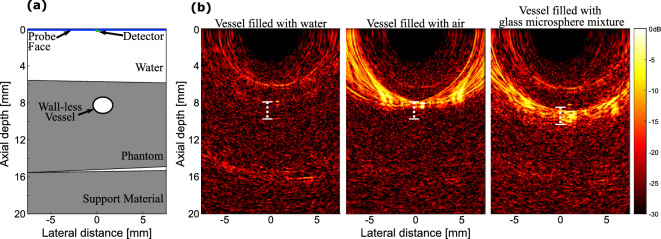
Freehand OpUS imaging of tissue-mimicking wall-less vessel phantom. (**a**) Schematic of the geometry of a tissue-mimicking vessel phantom, where the vessel was placed orthogonal to the image plane. The probe was placed at the surface of a water bath, and the phantom was supported by a separate block of tissue-mimicking material, a small amount of water filled the gaps between the two. (**b**) OpUS imaging of the wall-less vessel phantom when filled with water (left), air (middle), or contrast agent (right). Images displayed on a 30 dB logarithmic scale and normalised to the maximum value when the vessel was filled with microspheres. The position of the vessel lumen is marked in each frame. The video of the vessel phantom is available in supplementary video S1.

Based on the resolution maps (Fig. 4a,b), the lateral extent of the subsequent OpUS images was restricted to ± 7.5 mm around the centre of the imaging array. This provided a wider field of view than previously presented designs (± 5 mm in phantom and *in vivo* imaging)^21^, whilst ensuring that imaging was conducted in a region in which all OpUS sources would contribute to the reconstructed pixel values. Outside of the chosen 15 mm lateral imaging aperture, only sources at the edges of the OpUS array would contribute to the reconstructed pixel values, resulting in poor contrast, coupled with the demonstrated drop-off in resolution. The axial extent of subsequent images was generally restricted to 20 mm, primarily as a result of the significant decrease in axial resolution at depth (Fig. 4b), and due to the fact that the majority of intended imaging targets only contained structure above this depth. This represented a significant increase in imaging depth over previously reported devices, which had a maximum imaging depth of 12 mm^21^. Images were reconstructed using a delay and sum (DaS) beamformer at pixel dimensions 20 µm (lateral) by 25 µm (axial).

The low number of OpUS sources and regular source pitch resulted in significant residual grating lobe artefacts (the “wing-shaped” artefacts observed extending laterally from each point target in Fig.4d,e). At shallow imaging depths these artefacts are dominant and display similar contrast levels to the signal generated by the point target (< 5 mm depth in Fig 4e). The relative strength of the artefacts significantly reduces with imaging depth as the distance between the main beam and the first grating lobe increased as seen in Fig. 4e. The relative strength of the grating lobe artefacts was stronger than that seen with previously reported systems^21^, due to the regular spacing and higher source pitch.

### Tissue mimicking phantom imaging

The performance of the freehand OpUS imaging system was assessed using a tissue-mimicking phantom containing a wall-less vessel (Fig. 5). When this vessel was filled with water the interface between water and the phantom material at the top of the phantom, both the top and bottom walls of the vessel and the bottom of the phantom could be seen. With the vessel filled with air, only the top surface of the vessel is visible due to the high echogenicity of air, to such a degree that all other structures in the phantom are obscured (Fig. 5(b)). When filled with microsphere solution, the entire vessel lumen was visualised. However, in all three cases the image quality was limited by strong grating lobe artefacts due to the large source element pitch.

**Figure 6:**
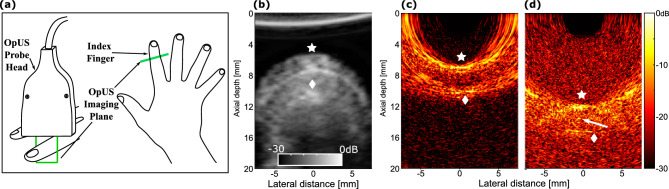
In vivo freehand OpUS imaging of the finger. (**a**) Schematic depiction of imaging geometry and OpUS probe placement used in volunteer study. (**b**) Conventional piezoelectric ultrasound image of the finger of a human volunteer, with skin and bone surfaces marked. (**c,d**) Freehand OpUS images of the index fingers of two different human volunteers at two depths. In (**b-d**) the skin surface is marked by $\star$ and the bone surface marked by $\diamond$, the additional reflection in (**d**) is marked with $\rightarrow$. Examples of finger imaging for different participants are available in supplementary video S2.

### *In Vivo* imaging

Finally, the clinical applicability was assessed through, to the authors’ knowledge, the first multi-participant *in vivo* freehand OpUS imaging study of healthy volunteers. Video rate imaging of the finger of each volunteer was used as an assessment of the function of the probe under controlled conditions, similar to those used to image tissue mimicking phantoms (Fig. 5), but with live tissue and finer internal structure than those developed in the phantoms (Fig. 6). Initial imaging of the fingers was conducted with the top of the finger at approximately the same depth as the vessel phantom surface, and in this position the top and bottom of the skin and the top of the finger bone could be clearly seen (Fig. 6c), and had contrasts (relative to water) of 19 ± 3 dB and 19 ± 4 dB respectively. Across all five participants the distance between skin and bone was 3.6 ± 0.5 mm, and remained consistent as the finger moved in the imaging space. These measurements are in line with reported dimensions in literature^27^ and when matched to conventional ultrasound measurements made of the same target. At this depth, the image was dominated by the strong reflection from the surface of the skin and any structure internal to the finger, apart from hard-reflecting bone was not visible. With the finger moved to greater depth more structure could be identified as the relative strength of the grating lobes is reduced (Fig. 6d), albeit at a lower contrast level. The skin surface curvature can be clearly seen, with a contrast of 16 ± 3 dB, as well as the bone surface, with a contrast of 14 ± 6 dB. A small additional reflection *ca.* 1.5 mm below the skin can be observed that generates a distinct set of grating lobes this is most likely the extensor tendon, or one of the digital blood vessels. This demonstrated effective OpUS imaging of an *in vivo* structure at up to 20 mm, effectively doubling the imaging depth presented previously, which was limited to structures that were at a maximum 10 mm from the probe face^21^. This was the first demonstration of a freehand OpUS probe imaging similar anatomical features in multiple participants, and demonstrated that the system would perform reliably, reproducibly, and yield relevant information in real-time and at video-rate.

**Figure 7:**
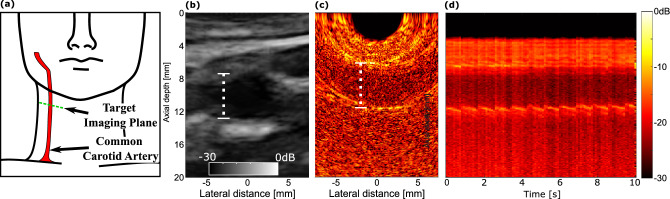
In vivo freehand OpUS imaging of the common carotid artery. (**a**) Schematic diagram of the target region for *in vivo* imaging of the common carotid artery. (**b**) Conventional piezoelectric ultrasound image of the common carotid of a human volunteer. (**c**) Freehand OpUS images of the common carotid artery *in vivo*, with the vessel running parallel to the imaging probe body. The vessel lumen is annotated in (**b,c**). (**d**) M-mode imaging of the common carotid artery *in vivo*, comprising laterally averaged signals, demonstrating the motion of the deeper wall of the artery over time. Examples of carotid artery imaging are shown in supplementary video S3.

To demonstrate the freehand OpUS system in a more clinically relevant setting, a final experiment was performed where the imaging probe was placed in direct contact with the skin of healthy volunteers using ultrasound coupling gel. *In vivo* imaging of the common carotid artery was chosen as it provided a simple imaging target with well defined position and visible motion under ultrasound imaging (Fig. 7, supplementary video S3). Imaging of the artery was attempted with the probe face both perpendicular and parallel to the artery. This process was performed on three out of the five study participants. To extract the heart rhythm of the participants, an M-mode image was generated by lateral spatial averaging of the centre 5 mm of the image, of which an example can be seen in Fig. 7. These M-mode images allowed for identification of the heartbeat which for three participants was found to be 71 beats per minute (BpM), ranging from 58 BpM to 93 BpM, the common carotid arteries were found to have an average axial thickness of 5.9 ± 0.9 mm and 5.6 ± 1.4 mm below the surface of the skin. These values sit within literature values^28^ for lumen diameter, demonstrating accurate extraction of physiological parameters. The width and depth of the artery in the imaging plane varied between volunteers, as a result of physiological differences and variable pressure applied with the probe. It should also be noted that the pressure applied with the probe was less than that conventionally applied with an electronic probe, so as to prevent mechanical damage to the detector fibre or the OpUS generating membrane. This resulted in lower transmission into the imaging volume. Despite this, the measured arteries demonstrated an average contrast of 16.3 ± 0.6 dB for the top of the lumen and 11 ± 2 dB for the bottom of the lumen, a 3 dB increase over previous reported *in vivo* contrast measurements^21^. In addition all vessels were measured at a greater depth than in previous work. The improved contrast enabled *in vivo* imaging at a higher dynamic range of 30 dB of the common carotid artery. For two participants the common carotid artery could not be visualised due to poor contrast. However, in these participants alternative anatomical features such as the branching point of the interior jugular vein were identified (see supplementary video S4).

## Discussion

In this work a novel freehand OpUS imaging system is presented that represents a significant step towards clinical translation of this technology. A ruggedised probe design and a mobile imaging console were combined to form an imaging platform approximating a commercial elctronic-based imaging system. The system presented was capable of real-time, video rate imaging without averaging at frame rates up to 24 Hz. Compared to previous freehand OpUS probes, this device was capable of imaging at greater penetration depths and higher frame rates, and was used for the first multi-participant *in vivo* imaging study with probes of this type.

A number of novel aspects in probe design were combined in the design of this device. Direct printing of a large array of eccentric optical waveguides provided a robust, reliable and scalable method of generating elliptical OpUS sources in a handheld device. The fabricated waveguide array demonstrated accurate source positioning and consistent source shaping across the array. The techniques developed to process large arrays of optic fibres using laser cutting and manual polishing simplified the fabrication process of the fibre bundles and allowed for flat-faced fibre optic arrays of varying geometries, without the need for precise cleaving of each fibre in the array, or accurate alignment approaches that require expensive commercial manufacture. The completed imaging probe, with the full 11 meter optical fibre bundle and protective clamshell, and strain relief pieces, enabled the system to be used in a more versatile freehand manner than previous probes without the risk of fibre damage, enabling the *in vivo* imaging presented here to be conducted rapidly and repeatedly. The probe also provided the first means of demonstrating OpUS imaging outside of the laboratory alongside other imaging modalities such as cone-beam computed tomography (CB-CT)^29^, in which the probe head was positioned at a significant distance from the imaging console during imaging.

Whilst the *in vivo* imaging conducted in this study was at a small scale, the ability to image the same structure in multiple volunteers indicates that freehand OpUS devices are capable of reliable imaging of a range of *in vivo* imaging targets, and could be used repeatedly over multiple experiments without impact on imaging quality. The *in vivo* imaging study demonstrated imaging of a number of anatomical features (demonstrated in supplementary videos S3 and S4) and confirmed that freehand OpUS could be applied in a practical fashion.

Many aspects of the system design presented that enabled the *in vivo* study also open up potential routes for clinical application. The extended fibre bundle length and metal-free probe head will enable the probe to be applied in a range of environments, particularly in magnetic resonance imaging (MRI) or computed tomography (CT) systems, where conventional piezoelectric devices exhibit strong interaction with the electromagnetic fields and induce image distortion and increased risk to the patient^30^. This multimodal imaging capability of fibre-optic OpUS imaging probes has been suggested previously^21^, and compatibility for imaging alongside CT has recently been demonstrated^29^. The video-rate imaging capabilities and good contrast of *in vivo* imaging targets indicate that freehand OpUS devices of this type could provide an alternative to current gating methodologies, monitoring the heartbeat of the patient during scans could provide a gating signal for cardiac MRI or CT and provide image data for concurrent imaging of relevant targets. With the identification of a suitable structure in OpUS this could also be used for breathing-cycle gating, enabling clearer pulmonary MRI or CT, without the need for breath-hold imaging which can often be uncomfortable for patients.

The system presented here was demonstrated to achieve effective imaging at depths of up to 30 mm of weakly echogenic wire targets, and imaging at up to 20 mm for tissue mimicking phantoms and *in vivo* tissues. The elliptical OpUS sources generated by this probe yielded higher penetration depths than those seen with previous handheld probes, however the source-spacing required by the underlying waveguide structure was far larger than the Nyquist limit for the central frequency of the probe, and the 3D printing method used prevented printing of the waveguides at a lower pitch. In turn, this limited the potential application of spatial apodisation schemes that have previously been used to overcome the impact of grating lobe artefacts^9,10^, as the central elements in the array would need to have a far lower pitch than that achievable with this printing method. As a result, the imaging was significantly impacted by strong grating lobe artefacts that limited the dynamic range of the images; this effect was particularly impactful at low imaging depths where due to source directivity fewer sources were actively insonicating the target structure. The imaging artefacts generated by the probe design are one of the main barriers to clinical application of this form of freehand OpUS probe, and will need to be reduced in future probe designs.

The 400 µm pitch of the sources in the presented OpUS probe was far above the Nyquist-limited source spacing for the bandwidths generated (approximately 70 µm for the centre frequency of this probe), and the resulting grating lobes are prominent and have a high signal level relative to the imaging target. Reducing the inter-element pitch would reduce the intensity of these grating lobes, and bring the lobes closer to the main lobe, thereby reducing their impact. Inter-element pitch could be reduced by utilising higher resolution 3D printing to print waveguides at lower pitch^31^, or moving to an alternative method of shaping the OpUS fields such as light shaping holographic diffusers^24^ or acoustic lenses, coupled with a reduced fibre spacing in the fibre optic array. Alternatively, an increase in the number of pulse-echo channels would increase the level of cross-channel averaging in reconstruction and thereby improve the SNR of the reconstructed images. The channel count of a freehand OpUS probe could be increased by adding more sources or multiple detectors. Reducing the pitch of the source array would increase the potential number of sources in the same aperture, achieving a higher channel count. The use of a single detector resulted in limited channel count–and consequently limited image SNR–as well as a spatially varying image contrast. In addition, specular reflectors are not effectively visualised using just a single detector. As such, improvements to the detector system used in freehand OpUS devices would also significantly advance the paradigm towards clinical relevance. Increasing the number of fibre-optic Fabry-Pérot detectors is currently impractical due to the associated system size and expense of adding an additional interrogation source and optical path, however utilisation of optical switches to interrogate multiple cavities or fibre-coupled planar cavities may provide an avenue to achieve multi-point ultrasound detection in a freehand probe. New methods for generating arrays of optical ultrasound detectors have recently been explored, with arrays of ring resonators^4,32^ providing multiple detector points with only a single wide-bandwidth light source.

Alternatively, moving to a different beamformer for image reconstruction could reduce the impact of the grating lobe artefacts, recently model-based inversion beamforming has been demonstrated to be effective in reducing such artefacts in low-channel-count OpUS devices, however this method has not been demonstrated to operate in real-time^33^. Non-linear beamformers such as delay multiply and sum (DMaS) have been demonstrated to be an effective method for improving the imaging quality of low channel count freehand OpUS devices in video-rate imaging^24^, however these methods impact appearance of the actual signal in the image, making the generated images challenging to interpret.

The freehand OpUS imaging probe and system here feature a ruggedised probe design free from metal and electronics, a compact and mobile acquisition system, and a long cable lead, allowing for versatile deployment in a wide range of scenarios, including electromagnetically harsh environments such as within MRI or CT scanners^29^. A multi-participant *in vivo* study demonstrated the reliability and repeatability of freehand OpUS imaging, and a frame rate of up to 24 Hz and imaging depth of up to 30 mm confirmed its clinical relevance and practicality. The freehand OpUS probe and accompanying imaging platform presented here thus represent an advance towards the clinical translation of this technology.

### Supplementary Information

Supplementary Information 1.

Supplementary Information 2.

Supplementary Information 3.

Supplementary Information 4.

Supplementary Information 5.

## Data Availability

OpUS probe characterisation and phantom imaging datasets are available upon reasonable request to the corresponding author, however *in vivo* data are not publicly available as participants did not consent to public data sharing.
