# Supplementary material for: Towards clinical application of freehand optical ultrasound imaging
Source: Sci Rep. 2024 Aug 13;14:18779. doi: 10.1038/s41598-024-69826-1 (PMC11322517; doi:10.1038/s41598-024-69826-1)
Supplement: Supplementary file 5 — Supplementary Information 5. [file 41598_2024_69826_MOESM5_ESM.pdf]

Towards Clinical Application of Freehand Optical Ultrasound Imaging: Supplementary Video Legend

Fraser T. Watt[1,2,*], Eleanor C. Mackle[1,2], Edward Z. Zhang[1,2], Paul C. Beard[1,2] , and Erwin J. Alles[1,2]
[1]Wellcome / EPSRC Centre for Interventional and Surgical Sciences, University College London, London, UK.
[2]Department of Medical Physics & Biomedical Engineering, University College London, London, UK
*fraser.watt.20@ucl.ac.uk

Supplementary video S1:

Delay and Sum (DaS) reconstructed freehand optical ultrasound (OpUS) video of a tissue-mimicking wall-less vessel phantom. The vessel starts out water filled, and at time point 00:04 a syringe filled with a water-glass microsphere mixture is compressed, expelling an air bubble into the vessel. This bubble clears at time point 00:05, when the vessel is refilled with the water-glass microsphere mixture, and the syringe remains at rest until the end of the video.

Supplementary video S2:

DaS reconstructed freehand OpUS video of *in vivo* imaging of the fingers of several study participants, demonstrating real-time capture of dynamic motion.
Time points of note:

- 00:03: participant 1, single finger moving in imaging field.
- 00:27: participant 1, moving hand reveals additional finger.
- 00:55: participant 2, single finger moving in imaging field.
- 1:21: participant 3, single finger moving in imaging plane, demonstrates internal reflecting structure between skin and bone surfaces.
- 1:28: participant 3, moving hand reveals additional finger.

Supplementary video S3:

DaS reconstructed freehand OpUS video of *in vivo* imaging of the common carotid artery of several *in vivo* study participants.
Time points of note:

- 00:01: participant 1, common carotid artery motion,
- 00:19: participant 2, arterial pulsatile motion, common carotid artery marked, possible shallow vessel also seen between 5mm and 7mm depth
- 00:30: participant 3, pulsatile motion, possible position of common carotid labelled.

Supplementary video S4:

DaS reconstructed freehand OpUS video of *in vivo* imaging of other structures in the neck.
Time points of note:

- 00:06: participant 1, branching vessel structures move into frame at approximately 14 mm depth. Direct comparison with structures imaged with conventional ultrasound imaging probe.
- 00:15: participant 1, probe motion brings one branch of vessel into clearer picture as second branch is moved out of frame
- 00:23: participant 2, non-pulsatile, strong reflecting structure with two distinct boundaries. Depth, geometry and approximate location suggests that this may be a strap muscle running parallel to the probe surface.
